# Development and Characterization of a Novel Congenital Acute Erythroid Leukemia Cell Line with Unique Features

**DOI:** 10.3390/cancers18091396

**Published:** 2026-04-28

**Authors:** Prasiksha Sitaula, Manisha Gadgeel, Holly Edwards, Lisa Polin, Juiwanna Kushner, Sijana H. Dzinic, Kathryn White, Yubin Ge, Jeffrey W. Taub, Katherine Gurdziel, Hunter Dlugas, Greg Dyson, Rozzelle Arlene, David Carr, Omar Moussa, Süreyya Savaşan

**Affiliations:** 1 Division of Hematology/Oncology, Children’s Hospital of Michigan, Detroit, MI 48201, USA; psitaula@anmed.org (P.S.); jtaub@med.wayne.edu (J.W.T.); 2 Division of Hematology/Oncology, Flow Cytometry Laboratory, Children’s Hospital of Michigan, Detroit, MI 48201, USA; mgadgee@med.wayne.edu; 3 Department of Oncology, Wayne State University School of Medicine, Detroit, MI 48201, USA; pitmanh@karmanos.org (H.E.); polinl@karmanos.org (L.P.); kushnerj@karmanos.org (J.K.); dzinics@karmanos.org (S.H.D.); whitek@karmanos.org (K.W.); gey@karmanos.org (Y.G.); fy7392@wayne.edu (H.D.); dysong@karmanos.org (G.D.); 4 Molecular Therapeutics Program, Barbara Ann Karmanos Cancer Institute, Wayne State University School of Medicine, Detroit, MI 48201, USA; 5 School of Medicine, Wayne State University, Detroit, MI 48201, USA; 6 Genome Sciences Core, Department of Pharmacology and IEHS, Wayne State University, Detroit, MI 48201, USA; gurdziel@wayne.edu; 7 Biostatistics & Bioinformatics Core, Barbara Ann Karmanos Cancer Institute, Detroit, MI 48201, USA; 8 Division of Plastic and Reconstructive Surgery, Children’s Hospital of Michigan, Detroit, MI 48201, USA; arozzelle@dmc.org; 9 Department of Pathology, Detroit Medical Center, Wayne State University, Detroit, MI 48201, USA; hg1057@wayne.edu (D.C.); omoussa@dmc.org (O.M.); 10 Pediatric Blood and Marrow Transplantation Program, Division of Hematology/Oncology, Barbara Ann Karmanos Cancer Center, Children’s Hospital of Michigan, Detroit, MI 48201, USA; 11 Department of Pediatrics, Central Michigan University College of Medicine, Mt Clemons, MI 48859, USA

**Keywords:** congenital leukemia, acute erythroid leukemia, extramedullary disease, ascitic fluid, cell line

## Abstract

Background: Acute erythroid leukemia (AEL) or AML-M6 predominantly affects older adults and is rare in childhood. Compared with other AML subtypes, AEL remains relatively understudied because of its rarity. We established LS-CHM, a novel AEL cell line derived from the ascitic fluid of a patient with congenital leukemia. Interestingly, leukemic cells persisted in the ascitic fluid even after successful eradication from the bone marrow and extramedullary sites. Method: Leukemia cells from the ascites fluid exhibited robust proliferation in culture independent of cytokine requirement and were further characterized by flow cytometric immunophenotyping, cytogenetics, cell cycle and doubling time analysis, colony formation, genome and RNA sequencing, myeloid gene next generation sequencing, and cytotoxicity analysis. Results: LS-CHM displayed CD36, partial CD235a, CD31, CD43, and CD71 expression and demonstrated in vitro robust growth and high sensitivity to chemotherapeutic agents. A PDX mouse model showed development of leukemia. Genomic analysis revealed a frameshift *BCOR* mutation in the absence of additional mutations and downregulated *TP53* expression with an exonic non-deleterious mutation. RNA sequencing of LS-CHM cells revealed upregulation of two cohesin complex genes, *RAD21* and *SMC3,* whose high levels are associated with hematopoietic stem cell differentiation into erythroid lineage. Conclusions: LS-CHM represents the first congenital AEL-derived cell line, in contrast to the predominantly adult-origin and often secondary erythroid leukemia cell lines available currently. Thus, LS-CHM provides a unique pediatric and extramedullary AEL model, expanding the existing spectrum of AEL cell lines and offering valuable opportunities for biologic and therapeutic investigations.

## Simple Summary

Acute erythroid leukemia (AEL) or AML-M6, an often a fatal type of acute myeloid leukemia, is primarily seen in older adults and rarely in children. AEL is relatively understudied because of its rarity compared to other subtypes. In this report, we describe the establishment and characterization of a new AEL cell line, LS-CHM, with unique features derived from ascitic fluid of a patient with congenital erythroid leukemia. LS-CHM with *BCOR* mutation, which is very uncommon in the pediatric age group, provides new insights into leukemogenesis as a novel and the first congenital AEL cell line and is a useful tool for further studies with potential therapeutic implications.

## 1. Introduction

Acute erythroid leukemia (AEL), also referred to as acute myeloid leukemia (AML)-M6, is a rare AML subtype, representing 1–5% of adult AML cases and less than 1% of pediatric AML cases with poor prognosis and a median survival of 2–3 months [1,2,3,4]. AEL results from impaired terminal differentiation and uncontrolled proliferation of dysplastic erythroid progenitor cells [4]. Adult AEL arises from prior myeloid neoplasms or therapy related and nearly all are associated with *TP53* mutation. In contrast, pediatric AEL occurs de novo, frequently lacks *TP53* mutation and has a different pathogenesis than adult AEL [5,6]. Due to its rarity, AEL is understudied, and the development of novel AEL cell lines is essential to improve research and therapeutic strategies [1,3,7]. Here, we describe LS-CHM, a newly established first congenital AEL cell line with a *BCOR* mutation derived from ascitic fluid from a patient with congenital AEL. Congenital AML itself is an extremely rare condition with dismal prognosis and an estimated incidence of 1–5 cases per million live births [8]. It frequently presents with prominent extramedullary involvement including hepatosplenomegaly, central nervous system infiltration and skin lesions—leukemia cutis, sometimes occurring in the absence of bone marrow involvement at initial presentation [9]. Skin lesions are seen in 93% of cases and sometimes present as cutaneous myeloid sarcoma prior to the diagnosis of AML with bone marrow involvement [10,11].

**Figure 1:**
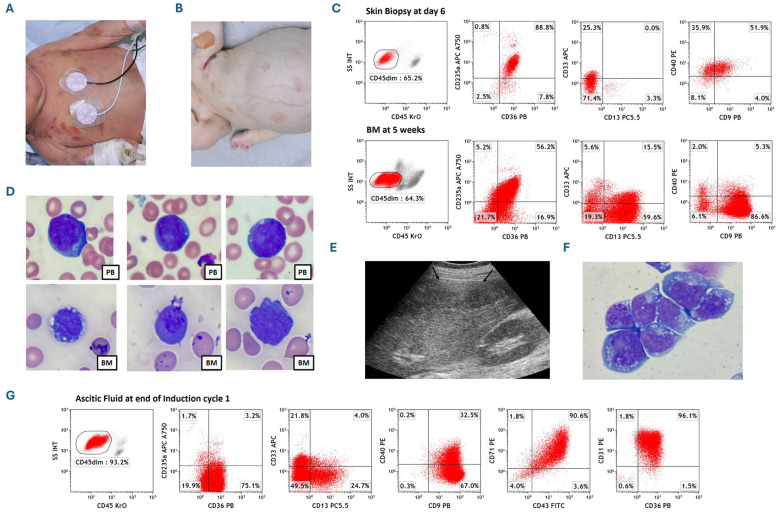
Presentation and course of the case. (**A**) Photograph depicts extensive erythematous cutaneous lesions in the newborn on the second day of life with congenital leukemia. (**B**) Photograph depicts significant abdominal distention and pallor along with some level of improvement in erythematous cutaneous lesions when the patient returned at 5 weeks of age. (**C**) Comparison of flow cytometric staining patterns of clonal cells obtained from skin biopsy and bone marrow. Skin biopsy dot-plots displaying leukemic population expressing CD235a, CD36, CD40, partial CD9 and CD33, while marrow cells expressing CD235a, CD36, CD9, CD13, and partial CD40 and CD33, indicating their erythroid lineage with minimal differences in two different tissue microenvironments. (**D**) Microscopic images of individual immature erythroid cells along with a single dysplastic normoblast in the middle lower row, observed on peripheral blood and bone marrow slides characterized by increased nucleocytoplasmic ratio, deep blue cytoplasm, and cytoplasmic vacuolation stained with Wright–Giemsa 100×. PB denotes peripheral blood; BM denotes bone marrow. (**E**) Abdominal ultrasound image showing ascites (arrowheads) after the first chemotherapy cycle. (**F**) Microscopic image of centrifuged ascites fluid leukemic cells characterized by irregular nuclear shape, blue cytoplasm, cytoplasmic vacuolation, and loose nuclear chromatin with several nucleoli with Wright–Giemsa staining 100×. (**G**) Flow cytometric dot-plots of AEL cells isolated from ascites fluid showing expression of CD36, CD9, CD31, CD43, and CD71, partial expression of CD13, CD33, and CD40, and very scant expression of CD235a.

The patient was a female infant born at 36 weeks of gestation with widespread cutaneous/subcutaneous, dusky red maculopapular/nodular lesions (Figure 1A). Initial tests showed a white blood count (WBC) of 9 × 10^9^/L, hemoglobin 12 g/dL, and platelets 92 × 10^9^/L. Peripheral blood (PB) flow cytometry showed 1% immature cells positive for CD235a, CD36, CD33, CD13, CD9, and CD40, and negative for CD34, CD117, CD38, CD41/61, and myeloperoxidase (MPO). Bone marrow (BM), cerebrospinal fluid (CSF), skin biopsies, and PB all showed immature cells with similar immunophenotypes and a normal female karyotype (46, XX). She was diagnosed with congenital AEL defined by differentiation, with only 2% BM involvement but significant extramedullary disease, with skin biopsy showing 65% erythroid blasts by flow cytometry (Figure 1B). Remarkably, there was self-involution of many cutaneous lesions and improved PB counts over three weeks without active therapy.

At five weeks, the patient presented with significant abdominal distension (Figure 1B). An abdominal ultrasound revealed enlarged abdominal lymph nodes. The WBC was elevated and flow cytometry showed BM blasts increased to 64% (Figure 1C) with a normal female karyotype and negative 56-myeloid gene next generation sequencing (NGS). Wright–Giemsa-stained PB and BM smears showed immature erythroid cells along with dysplastic normoblast characterized by increased nucleocytoplasmic ratio, deep blue cytoplasm, and cytoplasmic vacuolation (Figure 1D). At the end of the first induction chemotherapy with cytarabine, daunorubicin, and etoposide, and intrathecal therapy until CSF cleared, the BM was in remission with negative measurable residual disease (MRD). Abdominal ultrasound, however, showed large ascites and calcified lymph nodes (Figure 1E). Microscopic evaluation of Wright–Giemsa-stained ascites fluid leukemic cells displayed irregular nuclear shape, blue cytoplasm, cytoplasmic vacuolation, and loose nuclear chromatin with several nucleoli (Figure 1F). Cytogenetics of ascites fluid showed clonal evolution with trisomy 8 and 12 (47, XX, +8[12]/48, XX, +8, +12(3)/46, XX[5]). NGS panel showed *BCOR* mutation with three different clones: *BCOR* frameshift mutation (.c.1962dup,.p.Arg655GInfs*10) with VAF 37.4%, *BCOR* frameshift mutation (.c.1544_1569del,.p.Ser515Lysfs*33) VAF 2.7% and *BCOR* nonsense mutation (.c1573_1579del,.p.Met525*) with VAF 4.2%. Immunophenotyping showed 93% CD45-negative blasts that expressed CD36, CD9, CD31, CD71, and CD43, with scant expression of CD235a and partial expression of CD13, CD33 and CD40 (Figure 1G).

At the end of the second induction, the BM showed 2% MRD with karyotype 48, XX, +8, +12[6]/52, idem, +7, +8, +13, +19[5]/46, XX[9]. She could not proceed with the bone marrow transplant due to rapid clinical deterioration, which led to death at 4 months of age.

**Figure 2:**
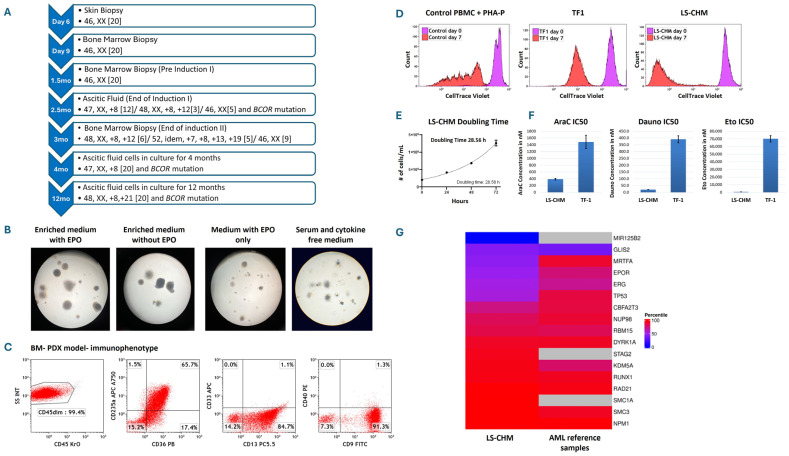
Course of cytogenetic/molecular changes in the primary cells and cultured cells and investigations for the LS-CHM AEL cell line establishment. (**A**) Comprehensive timeline of cytogenetic and molecular genetic changes in patient samples and culture-maintained leukemia cells. (**B**) Images of ascites fluid AEL cell colonies grown in different semi-solid culture media; MethoCult H4435-enriched methylcellulose medium for human cells with cytokines and erythropoietin (EPO), MethoCult H4535 enriched with cytokines without EPO, MethoCult H4330 medium with EPO only and Methocult H4236 serum-free methylcellulose medium without cytokines (StemCell Technologies, Vancouver, BC, Canada). Colony assays showed robust growth in cytokines supplemented media with or without EPO, while smaller colonies were observed in EPO-only and cytokine and serum-free media. (**C**) Flow cytometry dot-plots of bone marrow cells obtained from PDX mouse model developed by injecting ascites fluid AEL cells show similar phenotypic characteristics expressing CD235a, CD36, CD9, and CD13. Minor changes among expression patterns may be associated with the microenvironment leukemia cells. (**D**) CellTrace™ Violet assay conducted to evaluate proliferative potential of the cell line. Normal peripheral blood control lymphocytes treated with PHA-P exhibited five distinct cell divisions by day 7 compared to day 0. Conversely, both LS-CHM and TF1 cells display a single peak on day 7, indicating synchronized cell division throughout the entire cell population. The lower intensity of CellTrace™ Violet in LS-CHM cells on day 7 indicates a greater number of cell divisions occurred compared with TF1 cells. (**E**) Ascitic fluid leukemia cell doubling time. The LS-CHM AEL cells exhibited a short doubling time of 28.5 h (**F**) Evaluation and comparison of IC50 values for Cytarabine (AraC), Daunorubicin (Dauno) and Etoposide (Eto) between LS-CHM and TF1 cells. Lower IC50 values and higher drug sensitivity for all three drugs is seen in LS-CHM cells. (**G**) Comparison of gene expression rank percentile between LS-CHM sample from 4-week cultured cells RNA sequencing data and 45 pediatric AML reference samples. Each row of the heatmap corresponds to a gene of interest. The left and right columns depict the percentile of gene expression rank for our AEL sample and the average of 45 pediatric AML reference samples, respectively, ranging from blue (low expression) to red (high expression). The three gray boxes indicate that *SMC1A*, *STAG2,* and *MIR125B2* are not reported in the 45 pediatric reference samples.

After obtaining written informed consent from parents to use excess diagnostic material for research purposes, as approved by Central Michigan University Institutional Review Board, ascitic fluid cells were cryopreserved and later cultured in RPMI 1640 supplemented with 20% Fetal Bovine Serum (FBS) and gentamycin with and without cytokines at the density of 0.5 × 10^6^/mL at 37 °C and 5% CO_2_. Leukemia cells demonstrated continued rapid proliferation without cytokine support. The cultures were split every 2–3 days and maintained at the density of 0.5 to 1 × 10^6^/mL. Cytogenetics of cultured cells showed trisomy 8 in all mitoses at 4 months and further clonal evolution to 48, XX, +8, +21 at 12 months. This cytogenetic clonal evolution was acquired in vitro in culture environment (Figure 2A).

Myeloid gene NGS showed a *BCOR* frameshift mutation (c.1573_1579delATG; p.Met525*) with a variant allele frequency of 50%, reported as likely pathogenic, at 4 and 12 months in culture. The cells retained immunophenotypes consistent with AEL (CD45neg, CD235a partial+, CD36+, CD71+, CD31+) gaining partial CD235a expression and losing CD9, CD13, CD40, and CD33 expression at 12 months in culture compared to the diagnostic pattern and retained this immunophenotype later at different timepoints over 24 months in continuous culturing, confirming the cell type, origin and immunophenotypic stability (Supplementary Figure S1A). The cell line was designated LS-CHM.

## 2. Materials and Methods

### 2.1. Immunophenotyping of Cells Obtained from Skin Biopsy, Peripheral Blood, Bone Marrow, Cerebrospinal Fluid, and Ascites Fluid

Immunophenotyping for leukemia panel was performed on skin biopsy, peripheral blood, bone marrow, cerebrospinal fluid, and ascites fluid specimens to establish the diagnosis by staining the cells with an extensive panel of conjugated monoclonal antibodies: CD1a, CD2, CD3, CD4, CD5, CD7, CD8, CD9, CD10, CD11b, CD13, CD14, CD15, CD16, CD19, CD20, CD22, CD23, CD30, CD33, CD34, CD36, CD40, CD41+61, CD56, CD57, CD64, CD117, CD133, HLA-DR, and intracellular CD3, MPO, TdT, and CD79a (Beckman Coulter, Brea, CA, USA). Acquisition and analysis were performed on Beckman Coulter 10-color Gallios flow cytometer (Beckman Coulter, Brea, CA, USA).

### 2.2. Morphological Characterization of PB, BM and Ascites Fluid AEL Cells

PB and BM smears and cytospin slides of ascites fluid were stained with Wright–Giemsa-stain and examined under microscope for morphological characterization of leukemia cells.

### 2.3. In Vitro Culture of Leukemic Ascites Fluid Cells

Cryopreserved ascites fluid cells were thawed rapidly in 37 °C water bath. Cells were washed and half of the cells were resuspended in complete culture medium (RPMI +20% Fetal Bovine Serum + gentamycin), while the remaining aliquot was resuspended in complete culture medium supplemented with cytokines Stem Cell Factor (Peprotech, Rocky Hill, NJ, USA) (100 ng/mL), IL3 (10 ng/mL), IL6 (20 ng/mL), TPO (10 ng/mL), FLT3 ligand (10 ng/mL) and Erythropoietin (EPO) (Biolegend, San Diego, CA, USA) in 25 mL cell culture flasks at the concentration 0.5 × 10^6^/mL and allowed to grow in an incubator at 37 °C and 5% CO_2_ until a sufficient cell number was reached for cytotoxicity assay. Cultures were supplemented with their respective cell culture medium as needed to maintain cell concentration at 0.5 × 10^6^/mL. Cultures were later continued only in complete culture medium without any cytokines after noticing continued cell proliferation independent of cytokines.

### 2.4. Immunophenotypic Characterization of Culture Grown Leukemic Ascites Fluid Cells

Baseline immunophenotyping for leukemia panel and additional markers CD31, CD43, CD71 (Beckman Coulter, Brea, CA, USA) was performed after thawing the cryopreserved ascites fluid cells. Acquisition and analysis were performed on Beckman Coulter 10-color Gallios flow cytometer (Beckman Coulter, Brea, CA, USA). Immunophenotypic characterization of cultures with and without cytokines was assessed on day 3, 7 and 11. Immunophenotyping was also performed on passage 6 cells of the cultures grown without cytokines to observe any phenotypic changes. A 20% positivity cut-off level was used for individual markers.

### 2.5. Morphological Characterization of Culture Grown Cells

Cytospin slides were prepared and stained with Wright–Giemsa stain to assess the morphology of the culture grown cells.

### 2.6. Cytogenetic Analysis

Patient’s diagnostic and 6-week culture-grown ascites fluid cells were submitted to the Detroit Medical Center cytogenetics laboratory for chromosomal analysis. Twenty metaphase cells were analyzed, and karyotyping and Fluorescence In Situ Hybridization (FISH) analysis were performed.

### 2.7. Colony Forming Assay and Immunophenotypic Characterization of Colonies

Ascites fluid cells were cultured in four different semisolid culture media: MethoCult H4435 Enriched (methylcellulose medium for human cells with cytokines and with EPO), MethoCult H4535 Enriched without Erythropoietin (EPO) (methylcellulose medium for human cells with cytokines and without EPO) and MethoCult H4330 (methylcellulose medium for human cells with EPO only) and Methocult H4236 (serum-free methylcellulose medium without cytokines) for human cells (StemCell Technologies, Vancouver, BC, Canada) to determine colony formations in different growth environments. Cells were plated in 24-well culture plates at 500 cells/well in triplicates and cultured for 14 days at 37 °C and 5% CO_2_. The cells were then harvested from four different culture media and assessed for surface markers CD9, CD13, CD31, CD33, CD36, CD40, CD43, CD45, CD71, and CD235a. Acquisition and analysis was performed on Beckman Coulter 10-color Gallios flow cytometer (Beckman Coulter, Brea, CA, USA).

### 2.8. Doubling Time

Ascitic fluid cells were incubated at 2 × 10^5^ cells/mL in complete culture medium at 37 °C and 5% CO_2_. Cell counts were performed every 24 h to determine the doubling time.

### 2.9. Cell Trace Violet Assay

CellTrace Violet (Invitrogen, Waltham, MA, USA) 5 mM stock solution was prepared by dissolving the contents of one vial of CellTrace Violet in 20 µL DMSO. A total of 1 µL of 5 mM CellTrace violet was added to 2 × 10^6^ cells suspended in 1 mL PBS. Cells were incubated for 20 min at 37 °C and 5% CO_2_. After 20 min, 5 mL of complete culture medium was added to the cells and incubated for 5 min to quench any unbound dye. Cells were then centrifuged, pelleted, and resuspended in complete culture medium. Cells were incubated for 10 min before analysis to allow the CellTrace Violet to undergo acetate hydrolysis. Acquisition and analysis were performed on Beckman Coulter 10-color Gallios flow cytometer on days 0, 2, 4 and 7 to track the cell division by monitoring CellTrace Violet fluorescence. Propidium Iodide (PI) (Biolegend, San Diego, CA, USA) was used as a viability dye to gate on live cells.

### 2.10. Cell Cycle Analysis

Suspension cells grown in complete culture medium were harvested and washed in PBS and fixed in cold 80% ethanol by adding drop wise to the pellet while vortexing to ensure fixation of all cells and prevent clumping. Cells were fixed for at least 30 min at 4 °C. Cells were then washed 2 times in PBS and centrifuged at 1000 g. Supernatant was discarded, and cells were treated with PI/RNase to lyse free RNA and ensure only DNA staining. After 24 h incubation at 4 °C, acquisition and analysis were performed on Beckman Coulter Gallios flow cytometer to measure the PI fluorescence after excluding the doublets to determine percent populations in G0/G1, S and G2/M phase.

### 2.11. Dose Response Cytotoxicity Assay and IC_50_ Determination by Annexin V/PI Flow Cytometry Assay

A cytotoxicity assay with Annexin V and PI was performed on LS-CHM cells and established AEL cell line TF-1. LS-CHM cells were maintained in complete culture medium (RPMI-1640 supplemented with 20% fetal bovine serum + Gentamycin). As per growth requirements, TF-1 cells were maintained in complete culture medium supplemented with 2 ng/mL GMCSF (Biolegend, San Diego, CA, USA) at 37 °C and 5% CO_2_ in a humidified incubator. Cell viability prior to treatment was greater than 90%, as assessed by trypan blue exclusion.

Cytarabine, etoposide and Daunorubicin were prepared as a stock solution in PBS and diluted in complete culture medium immediately before use. Cells were seeded at 1 × 10^5^ cells/mL in 24-well plates and treated with six to eight increasing concentrations of the drug for 24 h. Untreated cells served as controls.

Following 24 h of incubation, cells were harvested, washed with cold PBS, and were stained with Annexin V FITC and PI and incubated for 15 min at room temperature in the dark, followed by the addition of Annexin buffer with counting beads prior to acquisition.

Samples were acquired on a 10-color Beckman Coulter Gallios flow cytometer (Beckman Coulter, Brea, CA, USA) and the absolute count of viable cells (Annexin V-/PI-) was recorded. Percent cytotoxicity was calculated as (control viable cell absolute count—drug-treated viable cell absolute count) × 100/control viable cell absolute count. The experiments were performed in triplicate. IC50 values were calculated with GraphPad Prism (version 10) using nonlinear regression analysis with [Inhibitor] vs. response–variable slope (four parameters) model. IC50 values for each drug were compared between TF-1 and LS-CHM.

### 2.12. Patient Derived Xenograft (PDX) Mouse Model

Three triple transgenic NSG-SGM3 (NSGS) mice (strain #013062; 6–8 weeks old and 23 g ± 2 g body weight at the time of implant; Jackson Laboratory, Bar Harbor, ME, USA) (1 × 10^6^ cells/mouse) were injected with primary leukemia cells via tail vein. Mice were euthanized when leukemic symptoms progressed (hindleg paralysis, >10–15% weight loss, internal mass > 500 mg), with all outcomes confirmed by necropsy. BM was later harvested for analysis. All mice were provided with food and water and libitum, given supportive fluids and supplements as needed, and housed within an Association for Assessment and Accreditation of Laboratory Animal Care (AAALAC) accredited animal facility with 24/7 veterinary care. In vivo experiments were approved by the Institutional Animal Care and Use Committee at Wayne State University.

### 2.13. RNA Sequencing

Four-week cultured cells were submitted for bulk RNA sequencing analysis at Genomics Science Core and Myeloid panel to Genetics and Molecular pathology laboratory, Wayne State University, Detroit, MI. Salmon was used to quantify RNA transcript abundance (i.e., expression), and RNA transcripts corresponding to protein-coding genes were used for further analysis [12]. Due to a sample size of one, no normalization was performed. The average gene expression of all genes reported in the 45 samples in the Children’s Oncology Group AML trials with bulk RNA-sequencing data available was used as a reference [13]. Specifically, for each gene reported in (i) our sample and (ii) the 45 pediatric AML reference samples, the rank of the average reported gene expression was computed.

### 2.14. Whole-Genome Sequencing

Four-week cultured cells were submitted for whole-genome sequencing analysis at the Genomics Science Core and Myeloid panel to Genetics and Molecular pathology laboratory, Wayne State University, Detroit, MI. A Burrows–Wheeler Aligner was used to align reads with the human genome (build 38), and short germline variants were determined using the Genome Analysis Toolkit’s Best Practice Workflow [14,15]. Low-quality variants with either (1) Phred-scaled probability of a homozygous-reference genotype less than 250, (2) Phred-scaled probability that the variant call is incorrect less than 90, (3) root mean square mapping quality of all reads spanning the given variant site less than 50, and/or (4) proportion of alternate allele depth less than 1/6 were removed using bcftools [16]. The tool ANNOVAR (June 2020 version) was used to annotate additional information such as the gene(s); each variant is found in [17].

### 2.15. Myeloid Next Generation Sequencing

BM at diagnosis and four- and twelve-month cultured cells were submitted for Myeloid gene panel to Genetics and Molecular pathology laboratory, Wayne State University, Detroit, MI. Multiplex PCR of 568 amplicons was performed using the Illumina TruSight Myeloid Panel to target frequently mutated regions of 49 genes implicated in AML, myelodysplastic syndromes, myeloproliferative neoplasms, chronic myelogenous leukemia, chronic myelomonocytic leukemia and juvenile myelomonocytic leukemia. Next generation sequencing was performed on Illumina’s MiSeqDx with 150 bp paired end reads and a mean depth of coverage of ≥1000×. Sequencing data was analyzed with Illumina’s Local Run Manager software using the published human genome build UCSC hg19 as the reference sequence. Agilent’s Alissa Interpret software was used to evaluate sequence changes in this individual. Reported sequence variants with the following features are confirmed by Sanger sequencing or another appropriate method: (1) variants with variant allele fractions less than 10%; (2) variants with variant allele fractions between 10 and 20% and read depth 500×. Mutation nomenclature is based on the recommendations of the Human Genome Variation Society (HGVS). Variants are classified according to the Association for Molecular Pathology, American Society of Clinical Oncology and College of American Pathologists standards and guidelines for the interpretation and reporting of sequence variants in [18].

### 2.16. Mycoplasma Testing of LS-CHM

Testing for the presence of mycoplasma in cell culture was performed using the PCR method at four weeks in culture [19].

### 2.17. LS-CHM Authentication

An archived PBMC sample and twenty-seven-month cultured cells were submitted for STR profiling and comparison.

## 3. Results

### 3.1. Surface Marker Expression

Immunophenotyping on peripheral blood, bone marrow, skin biopsy revealed CD45 dim blast positive for CD235a, CD36, CD33, CD13, CD9, and CD40, and negative for CD34, CD117, CD38, CD41/61, and myeloperoxidase (MPO) with minimal differences due to different tissue microenvironments. CD40, CD36, and CD235a are expressed in the cell surface of red cell precursors and its predominance in our case, along with negative MPO expression, strongly favored AEL.

### 3.2. Morphological Characterization of PB, BM and Ascites Fluid Leukemia Cells

Wright–Giemsa-stained PB and BM smears showed immature erythroid cells along with dysplastic normoblast, characterized by increased nucleocytoplasmic ratio, deep blue cytoplasm, and cytoplasmic vacuolation. Microscopic evaluation of Wright–Giemsa-stained ascites fluid leukemic cells also displayed irregular nuclear shape, blue cytoplasm, cytoplasmic vacuolation, and loose nuclear chromatin with several nucleoli consistent with immature erythroblasts.

### 3.3. In Vitro Culture of Leukemic Ascites Fluid Cells

The in vitro culture grown cells retained an immunophenotype consistent with AEL (CD45neg, CD235a partial+, CD36+, CD71+, and CD31+) gaining partial CD235a expression and losing CD9, CD13, CD40, and CD33 expression at 12 months in culture compared to the diagnostic pattern, and retained this immunophenotype later at different timepoints (over 24 months), confirming the cell type.

### 3.4. Colony-Forming Assay and Immunophenotypic Characterization of Colonies

LS-CHM cells showed robust colony formation in cytokine-supplemented media with or without EPO compared to media without cytokines, displaying EPO independence for colony formation (Figure 2B). Flow cytometry showed harvested cells from the colonies from different culture conditions expressed similar surface markers to suspension cultures. This indicated that the growth of the colonies was not dependent on the presence of cytokines.

### 3.5. Cytogenetics Analysis

Twenty to thirty mitotic figures were analyzed. Initial cytogenetic analysis of the peripheral blood and bone marrow sample revealed normal karyotype 46, XX. Cytogenetics of ascites fluid showed clonal evolution with trisomy 8 and 12 (47, XX, +8[12]/48, XX, +8, +12(3)/46, XX[5]). Cytogenetics of cultured cells showed trisomy 8 in all mitoses at 4 months and further clonal evolution to 48, XX, +8, +21 at 12 months.

### 3.6. PDX Mouse Model

A patient-derived xenograft (PDX) model was developed by injecting primary ascites-derived cells into the tail vain of three triple-transgenic NSG-SGM3 mice. Mice exhibited a significant weight loss by day 36 and a median survival of 41 days. Cells recovered from mouse bone marrow showed immunophenotype similar to patient’s BM leukemia cells with 99.9% involvement (Figure 2C). About ~2% of the cells also exhibited stem cell features with CD45−, CD34+, CD38−, and CD96+ immunophenotypes, supporting tumorigenic potential (Supplementary Figure S1B). The peritoneal space was not involved in the PDX mouse model, reflecting differences in tumor microenvironment.

### 3.7. Cell Trace Analysis

A Cell Trace Violet assay was performed to track cell proliferation and different generations of the cell population to determine the number of cell divisions in culture over 7 days. Control lymphocytes treated with phytohemagglutinin-P 5 μg/mL underwent five cell divisions at day 7 whereas both LS-CHM and another AEL cell line, TF1, showed a single peak at day 7, indicating synchronized cell division of the entire population (Figure 2D).

### 3.8. Doubling Time

LS-CHM cells exhibited a rapid doubling time of 28.5 h (Figure 2E). LS-CHM cells collected at 12-month culture period were found to have a similar doubling time of 26.15 h, exhibiting continued robust proliferation.

### 3.9. Cell Cycle Analysis

Cell cycle analysis by flow cytometry of LS-CHM cells treated with Propidium Iodide (PI)/RNase (Biolegend, San Diego, CA, USA) to determine percent populations in G0/G1, S and G2/M phase by measuring PI fluorescence revealed a high S-phase population (16–28%), consistent with rapid proliferation (Supplementary Figure S2A).

### 3.10. Cytotoxic Effects of Chemotherapeutic Agents of LS Cell Line

LS-CHM showed significantly higher sensitivity, with lower IC50 values, to all three drugs—Cytarabin, Etoposide and Daunorubicin—compared to the TF-1 cell line. IC50 of Cytarabin was 390 nM for LS-CHM vs. 1487 nM for TF-1. IC50 of Etoposide was 664 nM for LS-CHM vs. 70141 nM for TF-1 and IC50 of Daunorubicin was 21 nM for LS-CHM vs. 391 nM for TF-1. (Figure 2F) [20]. Their sensitivity likely reflects their congenital leukemia origin, being a newly established cell line with a rapid proliferation rate.

### 3.11. RNA Sequencing

RNA sequencing showed upregulation of two cohesin complex genes, *RAD21* and *SMC3*, compared to the 45 pediatric AML reference samples. The other two cohesion complex genes, *SMC1A* and *STAG2*, are among the most highly expressed genes in LS-CHM cells as well. While *KDM5A* is upregulated, *TP53*, *EPOR*, and *NUP98* are downregulated compared to average pediatric AML samples. Furthermore, *NPM1* is among the most highly expressed genes in both LS-CHM cells and in the average pediatric AML samples (Figure 2G).

### 3.12. Whole Genome Sequencing

The *BCOR* gene showed a high number of variants, including three exonic variants, one of which was predicted to be deleterious. Other genes with high variant counts, such as *RUNX1*, *ERG*, *DYRK1A*, *STAG2*, *TET2*, *DNMT3A*, and *CBFA2T3*, contained only non-coding changes that were not pathogenic. Exonic variants were also detected in *TP53*, *RBM15*, *MRTFA*, *GLIS2*, and *SMC3*; however, none were classified as deleterious, highlighting *BCOR* as the only gene with a pathogenic mutation (Table 1).

### 3.13. Myeloid Next Generation Sequencing

At the end of induction, NGS results of primary ascites fluid cells demonstrated *BCOR* mutations with three distinct clones at differing variant allele frequencies (VAFs). A dominant clone with frameshift mutation (.c.1962dup, .p.Arg655GInfs*10, VAF 37.4%) and two smaller subclones, frameshift mutation (.c.1544_1569del, .p. Ser515Lysfs*33, VAF 2.7%) and nonsense mutation (.c1573_1579del, .p.Met525*, VAF 4.2%). In contrast, the myeloid NGS of ascites-derived cells maintained in culture at 4 and 12-month showed exclusive persistence of *BCOR* (c.1573_1579delATG; p.Met525*) with an increased VAF of 50%. There were no other significant mutations found including *TP53* variants.

### 3.14. Mycoplasma Testing

LS-CHM did not show any mycoplasma contamination as per PCR testing.

### 3.15. Authentication of LS-CHM

STR profiling of culture grown LS-CHM cells at 27 months and primary PBMC showed 100% match showing that the cell line is derived from the patient’s leukemia cells.

### 3.16. Cell Line Availability

This cell line will be available to the qualified investigators on reasonable request to the corresponding author.

## 4. Discussion

Establishing cell lines from primary AEL cells has been very challenging. We successfully established an LS-CHM cell line that has continued to proliferate in culture for more than two years while maintaining its morphology, doubling time, immunophenotype and cytogenetic characteristics.

An interesting feature of the LS-CHM cells is their ascitic fluid origin when leukemia cells were successfully eradicated from the bone marrow and several extramedullary sites. This raises the possibilities of the limited diffusion of chemotherapy into the peritoneal fluid, seeding from incomplete response in abdominal lymph nodes, or tissue tropism, as in vitro cytotoxicity assay and determination of IC50 for cytarabine, etoposide and daunorubicin showed LS-CHM having high sensitivity to all three drugs compared to the other established erythroid leukemia cell line TF-1.

LS-CHM and F-36P are both derived from effusion fluids (Table 2), indicating high proliferative and infiltrative behavior. This distinguishes them from other bone marrow-derived cell lines. LS-CHM proliferates faster than most other AEL cell lines, consistent with aggressive in vitro growth without cytokines with a doubling time of 28.5 h. In contrast, other erythroleukemia cell lines, including TF-1, AS-E2 and F-36P, display variable dependence on EPO, GM-CSF, or IL-3 [21,22,23,24,25,26,27,28,29].

Immunophenotypically, LS-CHM expresses CD31, CD36, CD43, and CD71, with partial CD235a and absence of lymphoid, myeloid, megakaryocytic, and stem cell markers (CD3, CD13, CD14, CD15, CD19, CD34, CD41, and CD117) defining an erythroid profile. In contrast, most other erythroid leukemia cell lines except AS-E2 express a broader myeloid, megakaryocytic or stem cell marker profile, including expression of the HLA-DR profile in addition to erythroid markers CD36, CD71 and CD235a [21,22,23,24,25,26,28,29]. Similar to the AS-E2 cell line, LS-CHM displays some late erythroid cell characteristics; however, it is highly proliferative and cytokine-independent [26].

RNA sequencing of LS-CHM cells at 4 weeks of culture revealed upregulation of two cohesin complex genes, *RAD21* and *SMC3*, compared to 45 pediatric AML reference samples. High cohesion levels are associated with hematopoietic stem cell differentiation into erythroid lineage [30,31]. The other two cohesion complex genes, *SMC1A* and *STAG2*, are among the most highly expressed genes in LS-CHM cells as well (Figure 2G). Furthermore, one of the cohesion complex genes, *SMC3*, contained exonic mutations and was observed in 50% of the cases with myeloid leukemia of Down syndrome in which the expression of erythroid-specific genes was observed (Table 1) [32,33,34]. While *KDM5A* is upregulated, *TP53*, *EPOR*, and *NUP98* are downregulated compared to average pediatric AML samples. Furthermore, *NPM1* is among the most highly expressed genes in both LS-CHM cells and the average pediatric AML samples. While these results are noteworthy, we caution against placing too much merit in them and advise they be treated more as preliminary results given our sample size of one.

LS-CHM is the first congenital AEL cell line, with the *BCOR* mutation suggesting a distinct leukemogenesis mechanism, contrasting with many adult-derived erythroleukemia cell lines, such as HEL, OCI-M1, AS-E2, F-36P, TF1, and a pediatric KMOE-2, which mainly feature *TP53* and *JAK2/NRAS/*KMT2A pathway mutations (Table 2). The *BCOR* gene is located on the X chromosome and plays a role in the repression of *BCL-6* transcription. It is a transcription factor involved in hematopoiesis. *BCOR* mutations are rarely found in pediatric AML (<2% cases) and are often acquired during disease progression [35]. The clinical significance of *BCOR* mutations in pediatric AML cases is unknown and requires further investigation [36]. *BCOR* mutations are detected in 4% of cytogenetically normal AML and are more commonly found in secondary AML than de novo AML in adult patients [37,38]. *BCOR* deficiency has been implicated in AEL development and associated with adverse outcome [39,40]. Sporotoletti et al. have shown that concomitant *BCOR* and *DNMT3A* loss leads to the development of AEL in mice [41]. LS-CHM harbors *BCOR* mutation alone. At 4 months in culture LS-CHM cells showed exclusive persistence of minor *BCOR* subclone (c.1573_1579delATG; p.Met525*) from primary ascites cells with an increased VAF of 50%, indicating selective outgrowth of previously minor *BCOR* mutant subclone in cultured conditions and loss of dominant clone (c.1962dup; p.Arg655Glnfs10, VAF 37.4%) observed in the primary cells. These findings may indicate culture-induced clonal selection in the absence of a native tumor microenvironment. At this time point, the LS-CHM cells cytogenetically showed trisomy 8 in all mitoses (47, XX, +8[20]) and later developed trisomy 21 (48, XX, +8, +21[20]) at 12 months in culture. The *BCOR* nonsense variant, p.Met525*, seen in LS-CHM, has not been previously reported. However, other genes critical to AML development that are often found concomitant with *BCOR* mutations, *DNMT3A*, *RUNX1*, *TET2,* and *GATA1*, were not affected [40]. Downregulation of *TP53* and a non-deleterious exonic mutation in *TP53* were also found in LS-CHM cells. Because spurious mutations can arise during the culturing process, there is a chance that some of the variants described were not present at the time of diagnosis [42].

## 5. Conclusions

Our case displayed the aggressive nature of congenital AEL, as the patient died at 4 months despite the treatment. These cases remain poorly understood because of the rarity of the disease and lack of representative experimental models. Pediatric AEL differs from adult-onset AEL in pathogenesis. This highlights an urgent need for developing in vitro translational models of pediatric AEL. The combination of congenital origin, ascitic fluid derivation, robust in vitro cytokine-independent growth, and the presence of a frameshift *BCOR* mutation with downregulated *TP53* expression and absence of co-operating mutations defines LS-CHM as the novel congenital AEL cell line. Distinct from adult-derived AEL cell lines that typically harbor *TP53*, *JAK2*, *NRAS*, or *KMT2A* alterations, LS-CHM with *BCOR* mutation and the absence of *TP53* mutation constitutes a valuable model for studying the biology of AEL with potential new insights into leukemogenesis and potential therapeutic implications [43,44].

## Figures and Tables

**Table 1:** LS-CHM whole genome sequencing data showing the number of variants in genes of interest that are commonly mutated in AML.

Gene	Number of Variants		
	All	Exonic	Deleterious Exonic
*TP53*	22	1	0
*RBM15*	19	2	0
*MRTFA*	147	2	0
*CBFA2T3*	250	0	0
*GLIS2*	20	1	0
*NUP98*	49	0	0
*KDM5A*	113	0	0
*ERG*	650	0	0
*RUNX1*	776	0	0
*DYRK1A*	571	0	0
*MIR125B2*	0	0	0
*SMC1A*	33	0	0
*SMC3*	294	3	0
*EPOR*	3	0	0
*RAD21*	159	0	0
*STAG2*	459	0	0
*NPM1*	94	0	0
*DNMT3A*	201	0	0
*BCOR*	305	3	1
*TET2*	438	0	0
*GATA1*	6	0	0

**Table 2:** Comparison of LS-CHM with commercially available established AEL cell lines.

Cell Line	Tissue of Origin	Sex, Age	Mutation	Doubling Time	Cytokine Dependence	Immunophenotype
LS-CHM	Ascitic fluid	Female, 5 weeks	*BCOR* (c.1573_1579delATG; p.Met525*)	28.5 h	Cytokine independent	CD3−, CD13−, CD14−, CD15−, CD19−, CD31+, CD34−, CD36+, CD41−, CD43+, CD71+, CD117−, CD235a partial+, HLA-DR−
HEL	Bone marrow	Male, 30 years	*JAK2*; p.Val617Phe (c.1849G>T); *TP53*; p.Met133Lys (c.398T>A)	36 h	Cytokine independent	CD3−, CD13+, CD14−, CD19−, CD33+, CD41a+, CD71+, CD235a+, HLA-DR+
TF-1	Peripheral blood	Male, 35 years	*BUB1-MGAT4A*, *CBFA2T3-ABHD12*, *NRAS* p.Gln61Pro (c.182A>C), *TP53* p.Ile251Thrfs*94 (c.752delT)	70 h	GM-CSF or IL-3 dependent	CD3−, CD13+, CD14−, CD15−, CD19−, CD33+, CD34+, CD41+, CD42+, CD71+, CD235a+, HLA-DR+
KMOE-2	Peripheral blood	Female, 2 years	*NRAS* p.Gln61Arg (c.182A>G); *TP53* p.Val272Met (c.814G>A)	24.2–~40–50 h across sources	cytokine independent	CD3−, CD13+, CD15−, CD19−, CD33+, CD34−, CD71+, CD235a−
OCI-M1	Leukemic blasts post-CLL treatment	62 years	TP53; p.Leu145Arg (c.434T>G); *TP53*; Simple; c.673-2A>T (IVS6-2A>T)	~30 h	Cytokine independent	CD3−, CD4+ CD13+, CD14−, CD15+, CD19−, CD33+, CD34−, CD41+, CD42−, CD71+, CD235+, HLA-DR+
AS-E2	Bone marrow	Male, 62 years	No data	49 h	EPO dependent	CD36+, CD41−, CD71+, CD235a+, HLA-DR−
F-36P	Pleural effusion	Male, 68 years	*KMT2A*; Simple; p.Glu766Gln (c.2296G>C); *TP53*; c.376-1G>A (p.Tyr126_Lys132del, c.376_396del21)	24–36 h	GM-CSF dependent	CD3−, CD13+, CD14−, CD15−, CD19−, CD33+, CD34+, CD41−, CD42−, CD71+, CD235a+

## Data Availability

The data that supports the findings of this study are available from the corresponding author upon reasonable request.

## Supplementary Materials

The following supporting information can be downloaded at: https://www.mdpi.com/article/10.3390/cancers18091396/s1, Figure S1: Immunophenotyping of LS-CHM cells after being maintained for 12 months in culture and leukemic stem cell marker profile of LS-CHM and the PDX mouse model; Figure S2: Repeat experiments on LS-CHM cells after being maintained for 12 months in culture for comparison with previous test results. Repeat experiments on LS-CHM cells after being maintained for 12 months in culture to compare with previous test results.

## Abbreviations

The following abbreviations are used in this manuscript:

AEL	Acute erythroid leukemia
AML	Acute myeloid leukemia
WBC	White blood count
PB	Peripheral blood
BM	Bone marrow
MPO	Myeloperoxidase
CSF	Cerebrospinal fluid
NGS	Next Generation Sequencing
MRD	Measurable residual disease
EPO	Erythropoietin
PI	Propidium Iodide
PDX	Patient-derived xenograft
