# Supplementary material for: Development and Characterization of a Novel Congenital Acute Erythroid Leukemia Cell Line with Unique Features
Source: Cancers (Basel). 2026 Apr 28;18(9):1396. doi: 10.3390/cancers18091396 (PMC13162582; doi:10.3390/cancers18091396)
Supplement: Supplementary file 1 [file cancers-18-01396-s001.zip › cancers-4070140-supplementary.pdf]

Supplementary File

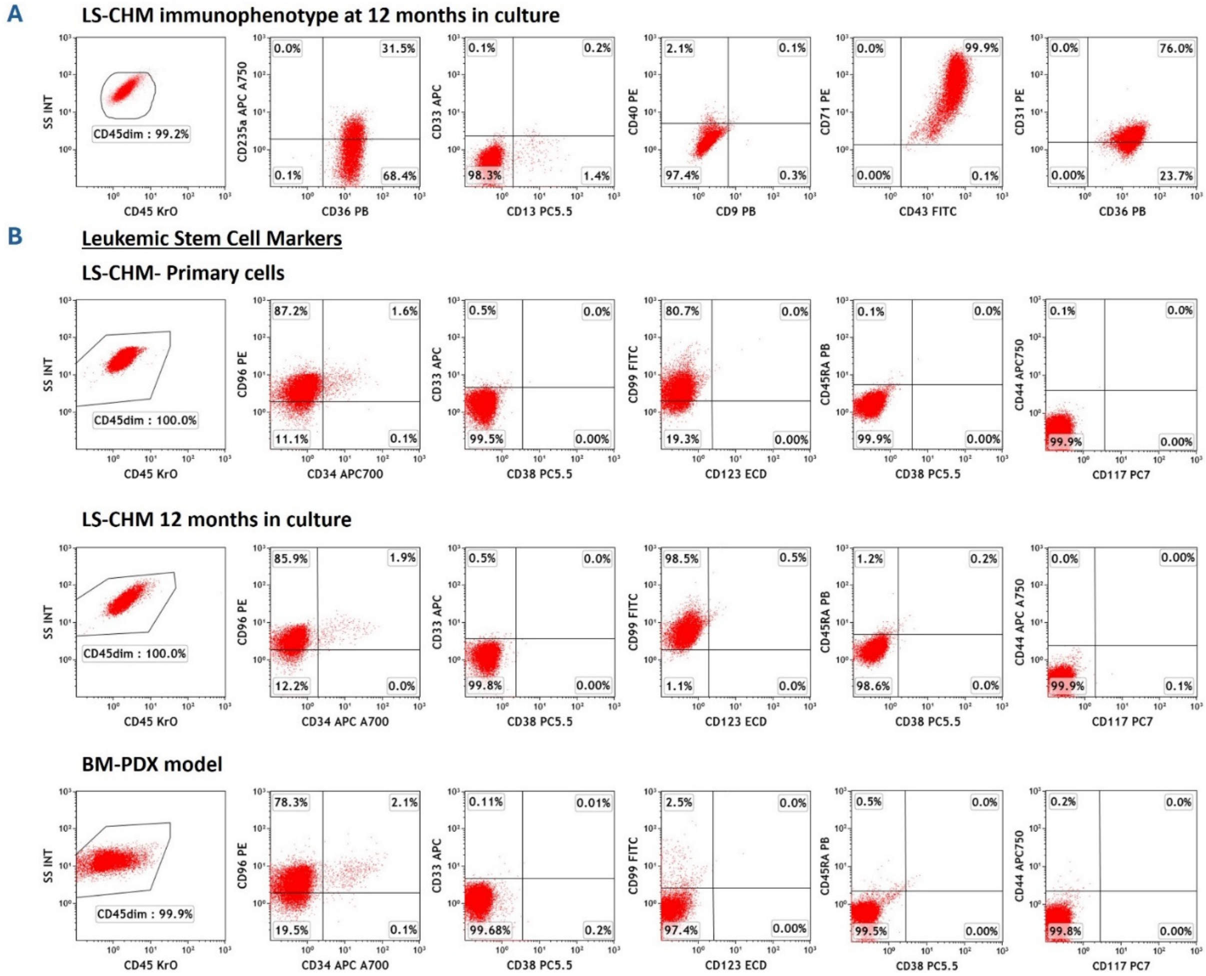

Supplementary Figure S1. Immunophenotyping of LS-CHM cells after being maintained for 12 months in the culture and Leukemic stem cell marker profile of LS-CHM and PDX mouse model. (A) Flow cytometric analysis of LS-CHM after 12 months in culture. Dot-plots showing expression of CD36, CD31, CD43, CD71, gain of partial expression of CD235a and loss of CD13, CD33, CD9, and CD40 expression. (B) Leukemic stem cell marker characterization of LS-CHM primary cells, cells at 12 months in culture, and cells obtained from PDX mouse model using CD38, CD34, CD123, CD96, CD99 and CD45RA. CD45– CD38– CD34+ CD96+ leukemic stem cells were 1.6%, 1.9% and 2.1% in primary AEL cells, LS-CHM cells maintained in the culture at 12 months and PDX mouse model, respectively.

A

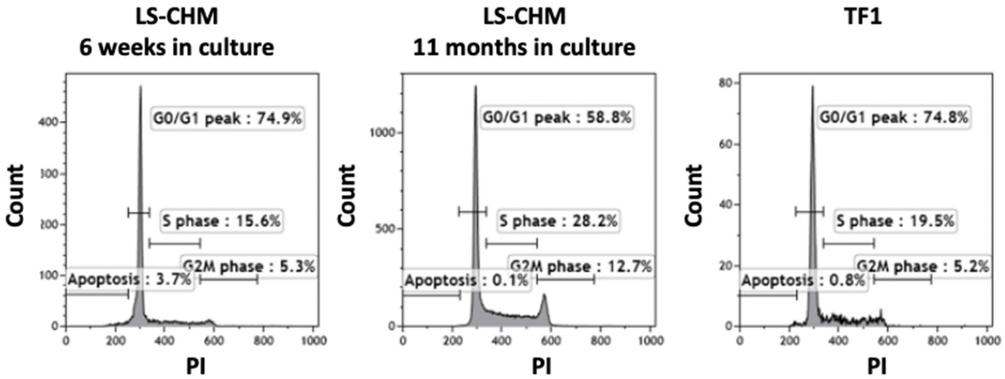

B

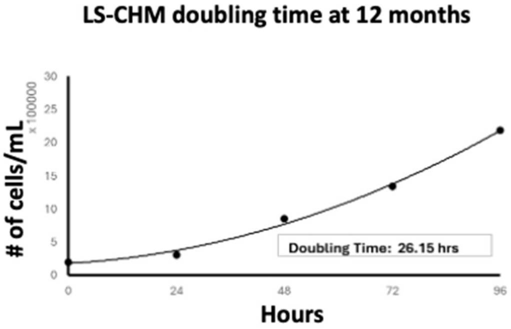

C

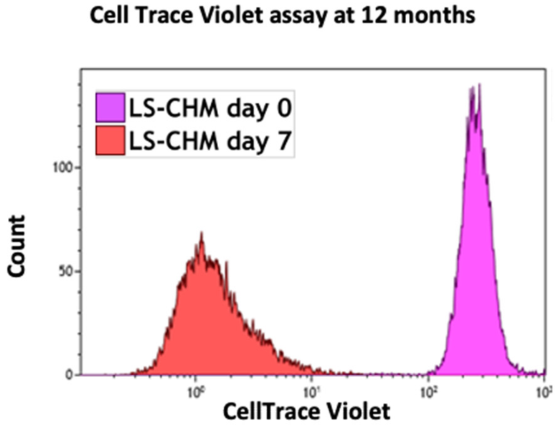

D

Karyotype: 48, XX, +8, +21 [cp20]					
Gene	cDNA	Protein	Depth	VAF	Classification
BCOR	c.1573_1579delATG	p.Met525*	9897	50%	Tier 2

Supplementary Figure S2. Repeat experiments on LS-CHM cells after being maintained for 12 months in the culture to compare with previous test results. (A) Flow cytometric cell cycle analysis of LS-CHM at 4 and 12 months in culture, and TF-1 cells showing with common significant representation of S phase population. (B) Doubling time of LS-CHM at 26.15 hours shows continued growth at the same rate after 12 months in continuous culture. (C) CellTrace™ Violet assay conducted to evaluate proliferative potential of the cell line shows a single peak at day 7, indicating synchronized cell division throughout the entire cell population similar to that seen in LS-CHM cells at 4 months in culture. (D) LS-CHM cells maintained in culture for 12 months showed trisomy 8 and trisomy 21 in all mitoses studied.
